# Enhanced RNA-Seq Expression Profiling and Functional Enrichment in Non-model Organisms Using Custom Annotations

**DOI:** 10.21769/BioProtoc.5555

**Published:** 2026-06-20

**Authors:** Infanta Saleth Teresa Eden M., Umashankar Vetrivel

**Affiliations:** 1 Department of Virology and Biotechnology, Bioinformatics Division, Indian Council for Medical Research -National Institute for Research in Tuberculosis (ICMR-NIRT), Chennai, India; 2 Academy of Scientific and Innovative Research (AcSIR), Ghaziabad-201002, Uttar Pradesh, India

**Keywords:** Gene enrichment analysis, Customized annotation, Non-model organisms, clusterProfiler, R packages

## Abstract

Functional enrichment analysis is essential for understanding the biological significance of differentially expressed genes. Commonly used tools such as g:Profiler, DAVID, and GOrilla are effective when applied to well-annotated model organisms. However, for non-model organisms, particularly for bacteria and other microorganisms, curated functional annotations are often scarce. In such cases, researchers often rely on homology-based approaches, using tools like BLAST to transfer annotations from closely related species. Although this strategy can yield some insights, it often introduces annotation errors and overlooks unique species-specific functions. To address this limitation, we present a user-friendly and adaptable method for creating custom annotation R packages using genomic data retrieved from NCBI. These packages can be directly imported as libraries into the R environment and are compatible with the clusterProfiler package, enabling effective gene ontology and pathway enrichment analysis. We demonstrate this approach by constructing an R annotation package for *Mycobacterium tuberculosis* H37Rv, as an example. The annotation package is then utilized to analyze differentially expressed genes from a subset of RNA-seq dataset (GSE292409), which investigates the transcriptional response of *M. tuberculosis* H37Rv to rifampicin treatment. The chosen dataset includes six samples, with three serving as untreated controls and three exposed to rifampicin for 1 h. Further, enrichment analysis was performed on genes to demonstrate changes in response to the treatment. This workflow provides a reliable and scalable solution for functional enrichment analysis in organisms with limited annotation resources. It also enhances the accuracy and biological relevance of gene expression interpretation in microbial genomics research.

Key features

• Comprehensive SQLite database with gene information and detailed annotation of all organisms in NCBI.

• Customized R annotation package built for *Mycobacterium tuberculosis* H37Rv by extracting species-specific records from the SQLite database using the taxonomic identifier.

• Gene ontology and KEGG enrichment analysis on significantly expressed genes from the RNA-seq dataset GSE292409 by importing the customized annotation package as an R library.

## Background

Gene enrichment analysis is a method to understand the functional pattern of a group of genes that are differentially expressed. While primary transcriptome analysis gives an overview of gene expression by quantifying RNA-Seq reads mapping to different genomic regions, gene enrichment analysis adds functional significance [1]. By aggregating signals from groups of genes, this method becomes critically important, as individual genes alone do not adequately reflect the functional dynamics of gene expression [2]. To date, there is a plethora of free resources available for defining the ontology and function based on statistical thresholds. These tools enable users to provide a list of genes and select the suitable organism and certain statistical parameters to design an ontology and pathway enrichment map. However, these tools often lack support for comprehensive, broad-spectrum analyses. Moreover, most do not allow seamless integration or reuse of results across different analytical workflows [3]. For larger datasets, issues pertaining to computational time and result accuracy also persist. Additionally, the need to rely on external tools for gene name conversion and result customization presents a further limitation.

Most gene ontology and pathway enrichment tools, such as g: Profiler, DAVID, GOrilla, BINGO, Enrichr, GOnet, ShinyGO, and KOBAS, provide enrichment analysis for well-annotated model organisms [4–11]. However, these tools offer limited support for organisms that are not commonly used as models, primarily due to the absence of comprehensive functional annotations. As a result, conducting enrichment analysis for such organisms remains a significant challenge. In many cases, when gene lists from these organisms are submitted, the tools may either reject the input or fail to return meaningful results. To overcome this limitation, researchers often follow a multi-step approach. This involves extracting the sequences of differentially expressed genes, identifying homologous sequences through similarity searches against reference databases of closely related species, and assigning gene ontology terms based on the best matching hits. The assigned ontology terms are then subjected to enrichment analysis using tools that accept ontology term input, since many platforms depend on gene identifiers from well-characterized species. This method has become a commonly used strategy to perform functional profiling in organisms for which detailed annotations are not available in standard gene ontology resources. Despite its practical use, there is no widely accepted protocol or standardized method for functional annotation and enrichment analysis in organisms that lack sufficient reference data. In an effort to address this gap, we explored open-source platforms, community discussions, and public repositories to identify reliable and accessible methods capable of producing biologically relevant results that are consistent with those obtained for model organisms. This search led us to the in-house development of a custom annotation strategy. We constructed organism-specific annotation packages using publicly available genomic data from NCBI and Expasy. These annotation packages can be imported as libraries into the R environment and are fully compatible with other established packages from Bioconductor. They allow researchers to perform gene ontology and pathway enrichment analysis with ease and flexibility [12,13]. We found this approach to enhance reproducibility and analytical depth, offering a dependable framework for functional analysis using well-established tools and customizable workflows.

## Software and datasets

**Table:** 

Type	Software/dataset/resource	Version	Date	License	Access (free or paid)
Data	RNA sequencing dataset (GEO ID: GSE292409), which captures the transcriptional response of various *Mycobacterium tuberculosis* strains following drug treatment at multiple time points. Out of 83 samples, 6 samples were chosen for demonstrative purposes. Three of these samples, which did not receive any drug treatment, and were designated as the control group. The remaining three samples were exposed to rifampicin for a duration of one hour and were categorized as the treatment group.	-	19-03-2025	-	Free
Software	SRA-Toolkit	3.2.1	18-03-2025	Public domain (United States Copyright Act)	Free
Software	FastQC	0.12.1	Released after 01-03-2023	GNU GENERAL PUBLIC LICENSE version 3	Free
Software	MultiQC	1.28	21-03-2025	GNU General Public License v3 (GPL-3.0-or-later)	Free
Software	TrimGalore	0.6.10	02-02-2023	GNU General Public License v3.0 (GPL-3.0)	Free
Software	Cutadapt	5.0	13-12-2024	MIT License	Free
Software	BWA	0.7.19-r1273	23-03-2025	GNU General Public License v3.0	Free
Software	samtools	1.21	12-09-2024	MIT License	Free
Software	featureCounts from Subread package	2.0.8	04-11-2024	MIT License	Free
Software	R	4.3.3	29-02-2024	GNU General Public License v3.0 (GPL-3.0)	Free
Software	AnnotationForge	1.44.0	24-10-2023	Artistic-2.0 license	Free
Software	clusterProfiler	4.10.1	08-03-2024	Artistic-2.0 license	Free
Software	ggplot2	3.5.1	22-04-2024	MIT + file LICENSE	Free
Operating system	Linux, Ubuntu (64-)	20.04.6	23-03-2023	GNU General Public License (GPL)	Free
Hardware	CPU: 24 core dual CPU (Intel^®^ Xeon(R) Gold 6240R) RAM: 512 GB Storage: 2 TB SSD recommended for generating the SQLite database and intermediate files used in the construction of the R annotation package Network: High-speed internet recommended for downloading large datasets	-	-	-	-

## Procedure

**Pseudocode for the steps used in the analysis:**

START OF ANALYSIS

# STEP 1: Download datasets from NCBI SRA and perform transcriptome analysis:

DOWNLOAD SRA datasets from NCBI using the commands: prefetch <sra_id> and fasterq-dump <sra_id>

PERFORM Initial QC of raw RNA-Seq reads using ‘fastqc’

PERFORM adapter trimming of raw RNA-Seq reads using ‘trimgalore’

PERFORM mapping of adapter-trimmed reads to Mtb H37Rv genome using ‘bwa’

PERFORM quantification of mapped bacterial mRNA reads using ‘featureCounts’

PERFORM DEG analysis from quantified reads using ‘DESeq2’ or ‘edgeR’

# STEP 2: Collecting gene information from NCBI and Expasy for constructing the R annotation package:

DOWNLOAD gene2accession.gz file from NCBI using ‘wget’

DOWNLOAD gene2go.gz file from NCBI using ‘wget’

DOWNLOAD gene2pubmed.gz file from NCBI using ‘wget’

DOWNLOAD gene2refseq.gz file from NCBI using ‘wget’

DOWNLOAD gene2ensembl.gz file from NCBI using ‘wget’

DOWNLOAD gene_info.gz file from NCBI using ‘wget’

DOWNLOAD idmapping_selected.tab.gz file from Expasy using ‘wget’

KEEP all downloaded files inside a folder

# STEP 3: Building customised SQLITE database using AnnotationForge R package:

START R environment

INSTALL R package AnnotationForge using Bioconductor

LOAD the library AnnotationForge into R

Build a reference NCBI.SQLite database containing all species for later species-specific data extraction.

BUILD your organism’s custom annotation R package from the source SQLite database using NCBI taxonomic identifier

IMPORT the customised package as an R library for enrichment analysis

# STEP 4: Perform Gene Enrichment and Pathway Analysis:

USE DEG results from the previous step for enrichment analysis

USE the enrichment results to draw enrichment plots and graphs

END OF ANALYSIS

**A. Downloading SRA datasets from NCBI**

The demonstrative transcriptome datasets (fastq files) were downloaded from NCBI SRA (data submitted by Bustad et al. [14]), wherein they have profiled the transcriptional response of different phylogenetically distinct strains of *Mycobacterium tuberculosis* by exposing them to isoniazid (INH) and rifampicin (RIF). The study comprises 83 sample runs, which are replicates of different Mtb strains subjected to drug treatments at different time points. Out of these runs, 6 samples belonging to the Mtb strain, H37Rv, were chosen, as the intention of this protocol is only to demonstrate the customized gene enrichment and pathway analysis. Among these 6 samples, 3 samples that were untreated with the drug were defined as the control group, and the remaining 3 samples that were treated with rifampicin for 1 h were defined as the treatment group. All these chosen samples are single-end reads (Table 1).

**Table 1.** List of the SRR sample IDs and type of treatment given for all 6 samples

SRR run IDs	Treatment (Mtb strain H37Rv subjected to drug treatment)
SRR32776059	Untreated (0 h)
SRR32776060	Untreated (0 h)
SRR32776061	Untreated (0 h)
SRR32776074	Rifampicin (1 h)
SRR32776075	Rifampicin (1 h)
SRR32776076	Rifampicin (1 h)

In the above table, the prefix SRR indicates the SRA Run Accession, an identifier format to define the names of individual samples that are deposited in the NCBI Sequence Read Archive (SRA).

**B. Transcriptome analysis for single-end RNA-Seq reads**

This section describes the processing steps applied to the raw FASTQ reads in order to identify differentially expressed genes. To perform transcriptome analysis of the bacterial RNA sequencing samples, follow the methodology outlined in our recent Bio-protocol publication [15]. After downloading the datasets from the NCBI repository, assess the initial quality of raw fastq reads using FastQC [16]. The quality assessment results for our demonstrative dataset are summarized in Table 2.

**Table 2.** Initial quality control statistics of raw fastq reads.

Sample name	% of duplicate reads	% GC content	Total number of sequences in millions
SRR32776059	64.207	64	7.0
SRR32776060	64.586	64	7.4
SRR32776061	67.908	63	8.8
SRR32776074	61.171	57	4.9
SRR32776075	56.439	58	1.4
SRR32776076	79.962	56	5.2

**1. Initial quality check on raw Fastq read files using FastQC**

$ prefetch SRR32776059 SRR32776060 SRR32776061 SRR32776074 SRR32776075 SRR32776076

$ fasterq-dump SRR32776059 SRR32776060 SRR32776061 SRR32776074 SRR32776075 SRR32776076

After running the above two commands, the raw fastq files will be generated for all 6 samples. Users must compress the files using gzip to reduce the storage space consumed by larger files before starting the analysis.

$ gzip *fastq

$ fastqc -t 10 *.fastq.gz

$ multiqc .

- where t is the number of threads to be used to run FastQC. The “.” in the multiqc command represents the current directory.

**2. Adapter trimming and final quality assessment of trimmed reads using TrimGalore**

Following the initial quality assessment, the raw sequencing reads were found to contain the Illumina universal adapter sequence “AGATCGGAAGAGC” at the 3′ end. To ensure accurate downstream processing, remove the adapter sequences from the raw reads using the TrimGalore tool [17,18]. After trimming, perform a final quality check to confirm the successful removal of adapters and to verify the overall quality of the cleaned reads.

The raw data in compressed .fq.gz/.fastq.gz format are used as input files to perform the below adapter trimming step.

*Note: Users are advised to create a directory for trimmed reads, which can be named “2.Trimmed_reads.”*

$ trim_galore --quality 20 --stringency 7 SRR32776059.fastq.gz SRR32776060.fastq.gz SRR32776061.fastq.gz SRR32776074.fastq.gz SRR32776075.fastq.gz SRR32776076.fastq.gz –output_dir 2.Trimmed_reads/ --cores 2

The above trimming step will remove reads that are shorter than 20 bp. The number of reads containing adapters in each sample, as well as the number of reads retained for subsequent downstream analysis, are shown in Table 3. Table 4 shows the final quality assessment in the sample reads after trimming the adapters and low-quality bases.

**Table 3.** Number of reads with adapters and number of reads retained in the samples after trimming.

Sample name	Total number of reads	Number of reads with adapters	Number of reads with shorter length (<20 bp)	Total number of reads retained after trimming
SRR32776059	7,036,732	110,510	85,825	6,950,907
SRR32776060	7,450,311	130,922	103,359	7,346,952
SRR32776061	8,833,603	124,838	92,946	8,740,657
SRR32776074	4,941,616	27,334	17,246	4,924,370
SRR32776075	1,472,812	9,413	6,246	1,466,566
SRR32776076	5,248,877	106,657	98,526	5,150,351

**Table 4.** Final quality control statistics of adapter-trimmed fastq reads.

Sample name	% of duplicate reads	% GC content	Total number of sequences in millions
SRR32776059	63.780	64	6.9
SRR32776060	64.102	64	7.3
SRR32776061	67.580	63	8.7
SRR32776074	61.035	57	4.9
SRR32776075	56.263	58	1.4
SRR32776076	79.593	56	5.1

The trimming step successfully removed adapters and most of the low-quality bases from the raw reads. Few samples contained minor polyA and polyG stretches, which did not impact overall data quality. Therefore, all the samples are retained for downstream analysis.

**3. Mapping of adapter-trimmed reads to the Mtb H37Rv genome**

After adapter trimming, map the high-quality reads to *Mycobacterium tuberculosis* H37Rv reference genome (accession: GCF_000195955.2). The Mtb reference genome file can be downloaded from the following link: https://www.ncbi.nlm.nih.gov/datasets/genome/GCF_000195955.2/. Perform genome indexing for Mtb H37Rv and map the adapter-trimmed reads to the genome index using the BWA aligner [19].

*Note: Before proceeding further, users have to create two directories named “Mtb_genome” and “3.Read_Mapping_Pathogen.”*

# Indexing Mtb genome using BWA short-read aligner$ bwa index GCF_000195955.2_ASM19595v2_genomic.fna

# Mapping reads to the genome index

$ bwa mem Mtb_genome/GCF_000195955.2_ASM19595v2_genomic.fnaSRR32776059_trimmed.fq.gz -t 20 | samtools view -@15 -b -S | samtools sort -@15 -o ../3.Read_Mapping_Pathogen/SRR32776059.sorted.bam -O BAM

$ bwa mem GCF_000195955.2_ASM19595v2_genomic.fna

SRR32776060_trimmed.fq.gz -t 20 | samtools view -@15 -b -S | samtools sort -@15 -o ../3.Read_Mapping_Pathogen/SRR32776060.sorted.bam -O BAM

$ bwa mem GCF_000195955.2_ASM19595v2_genomic.fna SRR32776061_trimmed.fq.gz -t 20 | samtools view -@15 -b -S | samtools sort -@15 -o ../3.Read_Mapping_Pathogen/SRR32776061.sorted.bam -O BAM

$ bwa mem GCF_000195955.2_ASM19595v2_genomic.fna SRR32776074_trimmed.fq.gz -t 20 | samtools view -@15 -b -S | samtools sort -@15 -o ../3.Read_Mapping_Pathogen/SRR32776074.sorted.bam -O BAM

$ bwa mem GCF_000195955.2_ASM19595v2_genomic.fna SRR32776075_trimmed.fq.gz -t 20 | samtools view -@15 -b -S | samtools sort -@15 -o ../3.Read_Mapping_Pathogen/SRR32776075.sorted.bam -O BAM

$ bwa mem GCF_000195955.2_ASM19595v2_genomic.fna SRR32776076_trimmed.fq.gz -t 20 | samtools view -@15 -b -S | samtools sort -@15 -o ../3.Read_Mapping_Pathogen/SRR32776076.sorted.bam -O BAM

*Note: For eukaryotic species, hisat2 needs to be used for indexing the genome and mapping reads to the genome. In such cases, users can refer to the commands below:*

# Extracting splice sites and exons from the genome

extract_splice_sites.py genomic.gtf > splice_sites.tsv

extract_exons.py genomic.gtf > exons.tsv

# Genome indexing using hisat2

hisat2-build eukaryotic_genome.fna eukaryotic_genome -p 50 --ss splice_sites.tsv --exon exons.tsv

# Mapping reads to eukaryotic genome

hisat2 --dta -x eukaryotic_genome -U SRR32776059_trimmed.fq.gz –p 35 --summary-file SRR32776059_mapping_stats.txt | samtools view -@10 -b -S | samtools sort -@10 -o SRR32776059.sorted.bam -O BAM

**Table 5.** Percentage of mapped and unmapped reads.

Sample name	Total number of high-quality reads	Number of mapped reads	% of mapped reads	Number of unmapped reads	% of unmapped reads
SRR32776059	6,950,907	6,655,304	95.74	295,603	4.25
SRR32776060	7,346,952	7,071,670	96.25	275,282	3.89
SRR32776061	8,740,657	8,159,665	93.35	580,992	6.64
SRR32776074	4,924,370	3,710,198	75.34	1,214,172	24.65
SRR32776075	1,466,566	1,013,339	69.09	453,227	30.90
SRR32776076	5,150,351	2,602,034	50.52	2,548,317	49.47

Table 5 shows the percentage of mapped and unmapped reads after mapping the adapter-trimmed reads to the reference genome. The above results from Table 5 indicate that the untreated samples exhibit a higher mapping rate compared to the rifampicin-treated samples. A consistent pattern is observed here, which will be summarized in detail in the subsequent steps to identify the genes to which these reads are mapped.

**4. Quantification of read counts using featureCounts**

Perform quantification of the reads mapped to different regions in the genome using featureCounts [20] to identify the key genes involved.

*Note: Users can create a directory named “4.Read_Count_Quantification.”*

$ featureCounts -a Mtb_Genome/genomic.gff -t 'gene' -g 'Name' SRR32776059.sorted.bam SRR32776060.sorted.bam SRR32776061.sorted.bam

SRR32776074.sorted.bam SRR32776075.sorted.bam SRR32776076.sorted.bam -o 4.Read_Count_Quantification/Pathogen_Quantification_results_SE.tsv -O -T 40

*Note: For eukaryotic species, users can use the gtf file for quantification and the command below:*

featureCounts -a genomic.gtf -t 'exon' -g 'gene_id' SRR32776059.sorted.bam SRR32776060.sorted.bam SRR32776061.sorted.bam SRR32776074.sorted.bam SRR32776075.sorted.bam SRR32776076.sorted.bam -p -o Eukaryotic_quantification_results.tsv -O --countReadPairs -T 40

**5. Differential expression analysis using DESeq2**

To perform differential expression analysis, use the quantified gene expression data as input, which can be carried out using established tools such as DESeq2 and edgeR [21,22]. This analysis is essential for identifying biologically significant changes by comparing gene expression levels between different experimental conditions or sample groups. In this demonstrative protocol, the transcriptional response of *Mycobacterium tuberculosis* H37Rv to rifampicin treatment is assessed by comparing gene expression profiles of treated samples with those of untreated controls, implementing DESeq2. Differentially expressed genes are obtained based on the following significance threshold: log2FoldChange -0.5 to +0.5 with padj < 0.05. All downstream analyses are performed within the R programming environment.

*Note: Before proceeding further, ensure that the read count data and its corresponding metadata follow the format recommended below to avoid errors during DEG analysis.*

## Read count data

Read count file is a matrix of count data, where:

Row names correspond to gene names.

Column names correspond to sample IDs/sample names.

The rows and columns must be filled with read counts, wherein each number represents the number of read counts for a particular gene in a given sample.

After quantification using featureCounts, the following commands should be used to generate the read count matrix to arrive at the above required format.

$ cut -f1,7- 4.Read_Count_Quantification/Pathogen_Quantification_results_SE.tsv | grep -v "#" | sed 's/.sorted.bam//g' > 4.Read_Count_Quantification/Pathogen_Quantification_matrix_SE.tsv

The ideal representation of readcounts as readmatrix, as discussed, is given in Table 6.

**Table 6.** Read count matrix format for DEG analysis.

GeneID	SRR32776059	SRR32776060	SRR32776061	SRR32776074	SRR32776075	SRR32776076
dnaA	3609	3983	5071	3252	1031	1297
dnaN	1267	1337	1704	1136	436	546
recF	974	980	1366	499	156	206
Rv0004	687	611	797	188	45	81
gyrB	14530	15821	20792	8669	2399	3787

## Metadata

Metadata is a tab-separated file that contains information about the samples and their corresponding treatment or condition. Table 7 represents the recommended format for metadata.

**Table 7.** Metadata format for DEG analysis

Sample	Condition
SRR32776059	control_0hr
SRR32776060	control_0hr
SRR32776061	control_0hr
SRR32776074	rifampicin_1hr
SRR32776075	rifampicin_1hr
SRR32776076	rifampicin_1hr

***Important note:*** *It is crucial to ensure that the order of sample names in the rows of the metadata matches the order of column names in the read count matrix file. Also, ensure that the columns in the metadata file are separated by only a single tab and that the file does not contain any extra white spaces or newlines.*

**Code snippet for DEG analysis using DESeq2**

*# Install and load DESeq2 library in R:*

if (!require("BiocManager", quietly = TRUE))

install.packages("BiocManager")

BiocManager::install("DESeq2")

library(DESeq2)

*# Install and load the following libraries for visualising the results:*

install.packages("ggplot2")

*install.packages("RColorBrewer")*

*install*.packages("*pheatmap*")

if (!require("BiocManager", quietly = TRUE))

install.packages("BiocManager")

BiocManager::install("EnhancedVolcano")

library(ggplot2)

library(RColorBrewer)

library(pheatmap)

library(EnhancedVolcano)

*# Load read count data and sample metadata information:*

read_counts <- read.delim("Pathogen_Quantification_matrix_SE.tsv", sep = "\t", row.names=1, header=TRUE)

metadata <- read.delim("metadata.txt", sep = "\t", header=TRUE)

*# Create DESeq2 dataset object:*

dds <- DESeqDataSetFromMatrix(countData = read_counts,colData = metadata,design = ~ Condition)

*# Filter rows with lesser read counts:*

keep <- rowSums(counts(dds)) >= 10

dds <- dds[keep,]

*# Apply dispersion-normalization to the filtered DESeq2 dataset:*

vsd <- varianceStabilizingTransformation(dds, blind = FALSE)

*# PCA plot to visualise the sample dispersion:*

x <- plotPCA(vsd, "Condition")

*# Label the PCA plot with sample names:*

x + geom_label(aes(label = name))

*# DESeq step : For size-factor estimation:*

dds <- DESeq(dds)

*# In the above step, DESeq2 applies ‘median of ratios’ method of normalization, which normalizes the data for sequencing depth and RNA composition and this method does not account for gene length compared to other normalization methods like TPM/RPKM/FPKM.*

*# Perform pairwise-comparison between treatment and control group:*

res <- results(dds, contrast = c("Condition", "rifampicin_1hr", "control_0hr"))

*# Volcano Plot to visualise the differentially expressed genes for comparison - treatment versus control:*

EnhancedVolcano(res,lab=rownames(res),x='log2FoldChange',y='pvalue',title='Comparison : rifampicin_1hr versus control_0hr',subtitle='Differential Expression analysis using DESeq2',pCutoff=0.05,FCcutoff=0.5,pointSize=1,labSize=2.5,labFace = 'bold',legendLabSize = 9,legendIconSize = 2.7)

*# Apply p-adj method to the DEG analysis results:*

res1 <- results(dds,contrast=c("Condition","rifampicin_1hr","control_0hr"), alpha=0.05, lfcThreshold=0.5, pAdjustMethod="BH")

*# Sorting DEG results based on padj/pvalue and filtering the sorted results:*

*# if the number of padj rows > 25*,

resOrdered <- res1[order(res1$padj),]

resFiltered <- subset(resOrdered,resOrdered$padj < 0.05)

*# otherwise, sort results according to pvalue.*

resOrdered <- res1[order(res1$pvalue),]

resFiltered <- subset(resOrdered,resOrdered$pvalue < 0.05)

*# Prepare a matrix of normalized read counts from the raw read counts:*

normalized_counts <- counts(dds, normalized=TRUE)

*# After executing the above command, the normalized counts are extracted from the dds object which was created using the command*, dds <- DESeq(dds)

*# Use the padj/pvalue based filtered DEG results to extract the normalized read counts of the significantly expressed genes:*

mat = normalized_counts[match((rownames(resFiltered)),rownames(normalized_counts)),]

*# Set colours for plotting heatmap:*

colors <- colorRampPalette(rev(brewer.pal(11, "RdYlGn")))(255)

*# Select the top 100genes for plotting heatmap:*

top100_DEG <- mat[1:100, ]

*# Plotting heatmap for visualising the top 100 genes:*

pheatmap(top100_DEG, scale = "row", color = colors, cluster_rows = TRUE, show_rownames = TRUE, cluster_cols = TRUE, filename = "rifampicin_1hr_Vs_control_0hr_Heatmap-Top100Genes.png", width = 10, height = 20, fontsize_row = 18, fontsize_col = 18)

# Save the filtered and unfiltered DEG results as .csv files

write.csv(resFiltered, "rifampicin_1hr_Vs_control_0hr_DEG_results_filtered.csv")

write.csv(res,"rifampicin_1hr_Vs_control_0hr_DEG_results_unfiltered.csv")

Alternatively, users can use edgeR for differential expression analysis. edgeR will not be discussed in detail, as this protocol mainly intends to demonstrate the customized gene enrichment analysis.

**Figure 1.**
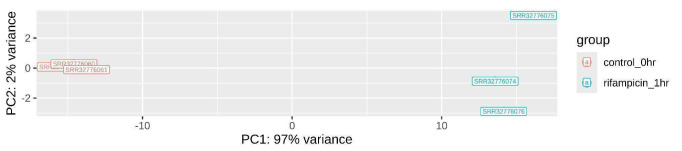
Principal component analysis of transcriptomic profiles. PC1 separates control (0 h; red) and rifampicin-treated (1 h; blue) samples. Replicates largely cluster by group, although one rifampicin replicate shows minor divergence along PC2.

**Figure 2.**
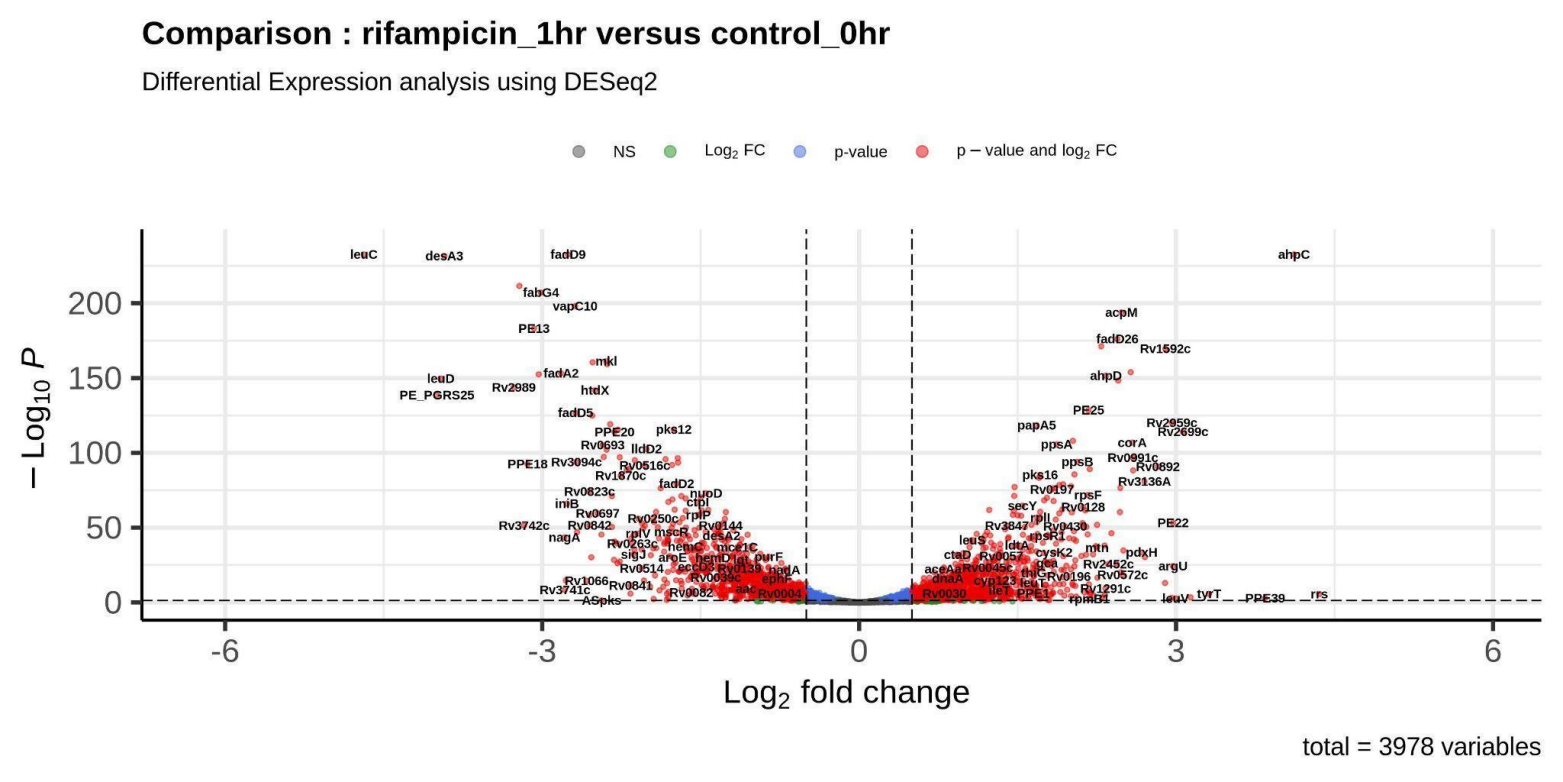
Volcano plot showing the differentially expressed genes (both upregulated genes toward the right side of the X-axis and downregulated genes toward the left side of the Y-axis). The above volcano plot is based on unadjusted p-values. Genes colored in red represent the differentially upregulated genes ≥ 0.5 (toward the right side of the X-axis) and differentially downregulated genes ≤ -0.5 (toward the left side of the X-axis).

**Figure 3.**
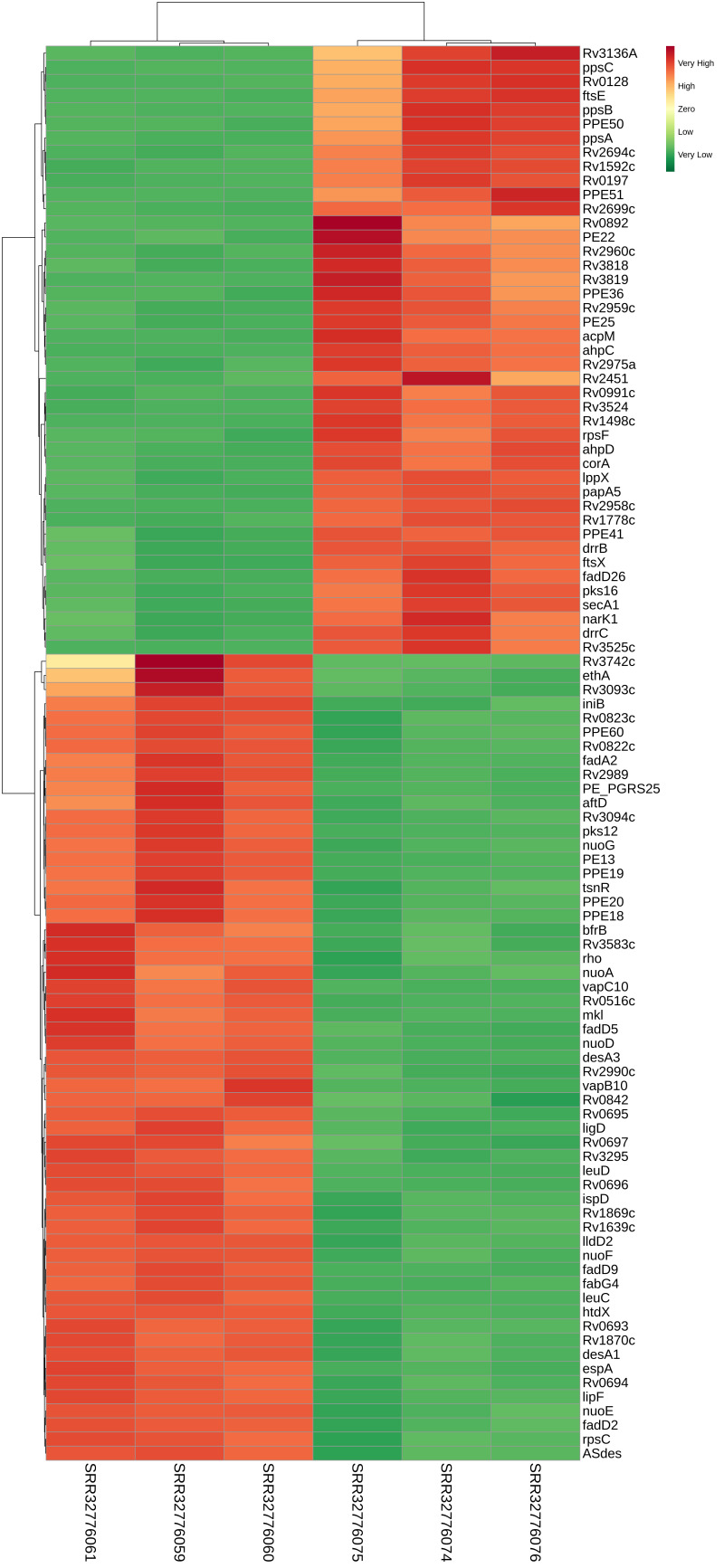
Heatmap showing the top 100 (50 up + 50 downregulated) differentially expressed genes in the treatment samples. The above heatmap can be inferred with the help of the color gradient strip at the top-right corner, which has a number scale ranging from -0.5 to +0.5. The heatmap is based on adjusted and filtered p-values (pAdjustMethod = BH; padj < 0.05). According to the gradient, gene slabs colored in yellow in the heatmap represent the neutrally expressed genes in that particular sample. Genes colored in orange and red represent the highly expressed genes, and those in green are less expressed genes in that sample group when compared to the adjacent sample group.

Figures 1–3 represent the results of differential expression analysis resulting from a comparison of rifampicin_1_hr_treated versus control_0_hr.

Differential expression analysis identified 3,978 genes in the comparison between rifampicin-treated (1 h) and control (0 h) samples. P-values are adjusted using the Benjamini–Hochberg method, and genes with an adjusted p-value (padj < 0.05) are considered significant. This yields 1,187 differentially expressed genes, of which 587 are upregulated, and 600 are downregulated. To predict and interpret the functional significance of the differentially expressed genes, perform subsequent analyses, including gene enrichment, gene ontology, and pathway enrichment.

**C. Gene enrichment for non-model organisms: Ontology and pathway analysis**

In this section, the functional roles of significantly expressed genes are explored by performing gene set enrichment and subsequently assessing their statistical significance. While several well-established open-source bioinformatics platforms support gene enrichment analysis for model organisms, such tools are scarce for non-model organisms. To address this gap, the following approach is demonstrated using the non-model organism *Mycobacterium tuberculosis* H37Rv, noting that the same protocol can be applied to any organism of choice. This method was adapted based on discussions within the Bioconductor open-source community [12,13]. This protocol will guide users in constructing customized, species-specific annotation R packages using resources from NCBI and ExPASy. The detailed steps of the construction of the annotation package and the execution of gene enrichment analysis will be discussed in the following sections.

**1. Construction of species-specific custom annotation package**

Before proceeding to the construction of the annotation package, it is essential to download and save the following files inside a single folder:

*# Create a folder name source_database:*

*# Enter into the folder : source_database*

*# Download the files using ‘wget’ command:*

wget https://ftp.ncbi.nlm.nih.gov/gene/DATA/gene2accession.gz

wget https://ftp.ncbi.nlm.nih.gov/gene/DATA/gene2go.gz

wget https://ftp.ncbi.nlm.nih.gov/gene/DATA/gene2pubmed.gz

wget https://ftp.ncbi.nlm.nih.gov/gene/DATA/gene2refseq.gz

wget https://ftp.ncbi.nlm.nih.gov/gene/DATA/gene2ensembl.gz

wget https://ftp.ncbi.nlm.nih.gov/gene/DATA/gene_info.gz

wget https://ftp.expasy.org/databases/uniprot/current_release/knowledgebase/idmapping/idmapping_selected.tab.gz

*# Open a Linux terminal. Start R session:*

*# Install R package - AnnotationForge:*

if (!require("BiocManager", quietly = TRUE))

install.packages("BiocManager")

BiocManager::install("AnnotationForge")

*# Load the library AnnotationForge:*

library(AnnotationForge)

*# Using the downloaded files, build source annotation SQLite database for all NCBI organisms:*

*(The following process requires connecting to ftp sites. Therefore, ensure that the internet connection is stable, and is not blocked by ftp firewall protection)*

*# Create an SQLite object to store the SQLite database:*

writeFilesToDb <- function(file, file.dir = ".") {

require("AnnotationForge", character.only = TRUE, quietly = TRUE)

require("RSQLite", character.only = TRUE, quietly = TRUE)

tmp <- file.path(file.dir, file)

pfiles <- AnnotationForge:::.primaryFiles()

file <- pfiles[file]

NCBIcon <- dbConnect(SQLite(), file.path(file.dir, "NCBI.sqlite"))

tableName <- sub(".gz","",names(file))

AnnotationForge:::.writeToNCBIDB(NCBIcon, tableName, filepath=tmp, file)

AnnotationForge:::.setNCBIDateStamp(NCBIcon, tableName)

dbDisconnect(NCBIcon)

}

*# List all the files which will be used to build the database:*

fls <- dir(".", "^gene.+gz")

*# Start to construct the database using the following command:*

*(This step generates a huge SQLite database file [~80-95GB file], which will be later sourced to build customised annotation packages for all non-model organisms)*

for(i in fls) writeFilesToDb(i)  *# This step is time-consuming*

*# After the above step runs successfully, the source SQLite file will be created inside the same directory with the name, ‘NCBI.sqlite’. Using this file, we will now create a custom annotation package for Mtb* H37Rv.

makeOrgPackageFromNCBI(version = '0.1', author = 'user <user@user.org>', maintainer = 'user', outputDir = '.', tax_id = '83332', genus = 'Mycobacterium', species = 'tuberculosis_H37Rv', rebuildCache = FALSE)

*# After the above command runs successfully, the custom annotation package for Mycobacterium tuberculosis* H37Rv *will be created inside a folder with the name ‘org.Mtuberculosis*H37Rv.eg.db’.

*# This custom annotation package can now be installed in R environment using the following command. Ensure the above created folder for the annotation package is found in the current working directory.*

install.packages("./org.*Mtuberculosis*H37Rv.*eg.db*", repos=NULL)

After this step, our customized annotation package can be simply loaded into the R environment for performing gene enrichment analysis.

**2. Gene enrichment and pathway analysis**

To perform gene enrichment analysis, use the DEG results obtained from the previous section. The analysis is carried out using clusterProfiler, a standalone R package widely used for functional characterization of both coding and non-coding elements across thousands of species. The package provides up-to-date annotations, supports multiple annotation methods for different analytical purposes, accepts diverse data formats, and offers flexible data manipulation along with extensive visualization options for efficient interpretation of results [23–26].

The custom annotation package, which was constructed for *Mycobacterium tuberculosis* H37Rv in the previous sections of this Bio-protocol, is imported as an R library and used in combination with clusterProfiler to perform enrichment analysis.

*# Start the R session. Load the following libraries:*

*# Install clusterProfiler using Bioconductor:*

if (!require("BiocManager", quietly = TRUE))

install.packages("BiocManager")

BiocManager::install("clusterProfiler")

*# Install enrichplot using Bioconductor:*

if (!require("BiocManager", quietly = TRUE))

install.packages("BiocManager")

BiocManager::install("enrichplot")

*# Install ggridges R package:*

install.packages("ggridges")

*# The custom organism annotation package was installed in the previous step. Therefore, we will now import the package into R:*

library(org.*Mtuberculosis*H37R*v.eg.db*)

*# Import other installed packages into R:*

library(clusterProfiler)

library(enrichplot)

library(ggridges)

*# We will now load the DEG results as input data for enrichment analysis:*

input <- read.table('rifampicin_1hr_Vs_control_0hr_DEG_results_filtered.csv',header=TRUE,row.names=1,sep=',')

*# Prepare a gene list from the above DEG results:*

gene_list <- row.names(input)

*# Extract log2FoldChange values for all the differentially expressed genes:*

original_gene_list <- input$log2FoldChange

*# Add the log2FoldChange information to the extracted gene list:*

names(original_gene_list) <- row.names(input)

*# Omit rows which have NA values:*

gene_list<-na.omit(original_gene_list)

*# Now, sort the gene list in decreasing order:*

gene_list = sort(gene_list, decreasing = TRUE)

*# Perform gene ontology analysis for Cellular Component:*

go_result_cc <- gseGO(geneList=gene_list, ont ='CC', keyType = 'SYMBOL', pvalueCutoff = 0.05, OrgDb = org.*Mtuberculosis*H37Rv.*eg.db*, verbose = TRUE, pAdjustMethod = 'BH', minGSSize = 3, maxGSSize = 800)

*# Perform gene ontology analysis for Biological Process:*

go_result_bp <- gseGO(geneList=gene_list, ont ='BP', keyType = 'SYMBOL', pvalueCutoff = 0.05, OrgDb = org.*Mtuberculosis*H37R*v.eg.db*, verbose = TRUE, pAdjustMethod = 'BH', minGSSize = 3, maxGSSize = 800)

*# Perform gene ontology analysis for Molecular Function:*

go_result_mf <- gseGO(geneList=gene_list, ont ='MF', keyType = 'SYMBOL', pvalueCutoff = 0.05, OrgDb = org.*Mtuberculosis*H37R*v.eg.db*, verbose = TRUE, pAdjustMethod = 'BH', minGSSize = 3, maxGSSize = 800)

*# Save the Gene Ontology results in .tsv files:*

write.table(go_result_cc@result,'Cellular_Component_GO_results.tsv')

write.table(go_result_bp@result,'Biological_Process_GO_results.tsv')

write.table(go_result_mf@result,'Molecular_Function_GO_results.tsv')

*# Now plot charts for visualising Gene Ontology results:*

*# Here, we write the plot visualisation scripts only for Cellular Component.*

*# In order to visualise plots for Biological Process and Molecular Function, repeat the below steps using go_result_bp and go_result_mf*

*# DOTPLOT:*

a <- dotplot(go_result_mf, showCategory = 5, split='.sign') + facet_grid(.~.sign) + theme(text = element_text(size = 22), axis.text = element_text(size = 22), legend.title = element_text(size = 22),axis.text.y=element_text(size = 22),axis.text.x=element_text(size = 22),axis.title.x=element_text(size = 22));

png('GO-Molecular_Function_dotplot.png', width = 1500, height = 2000, res = 140);

print(a);

dev.off()

a <- dotplot(go_result_bp, showCategory = 5, split='.sign') + facet_grid(.~.sign) + theme(text = element_text(size = 30), axis.text = element_text(size = 30), legend.title = element_text(size = 30),axis.text.y=element_text(size = 30),axis.text.x=element_text(size = 30),axis.title.x=element_text(size = 30));

png('GO-Biological_Process_dotplot.png', width = 2000, height = 2000, res = 140);

print(a);

dev.off()

*# ENRICHMENT MAP:*

x2 <- pairwise_termsim(go_result_mf);

b <- emapplot(x2, showCategory = 5, cex_label_category = 1.3) + theme(text = element_text(size = 20), legend.title=element_text(size = 20), legend.text=element_text(size = 20));

png('GO-Molecular_Function_enrichmentmap.png', width = 1500, height = 1000, res = 140);

print(b);

dev.off()

x2 <- pairwise_termsim(go_result_bp);

b <- emapplot(x2, showCategory = 5, cex_label_category = 1.3) + theme(text = element_text(size = 20), legend.title=element_text(size = 20), legend.text=element_text(size = 20));

png('GO-Biological_Process_enrichmentmap.png', width = 1500, height = 1000, res = 140);

print(b);

dev.off()

*# NETPLOT:*

c <- cnetplot(go_result_mf,categorySize = 'pvalue',foldChange = gene_list,showCategory = 3, cex_label_category = 1.3) + theme(text=element_text(size = 30),legend.title=element_text(size = 21), legend.text=element_text(size = 21));

png('GO-Molecular_Function_netplot.png', width = 3500, height = 2000, res = 200);

print(c);

dev.off()

c <- cnetplot(go_result_bp,categorySize = 'pvalue',foldChange = gene_list,showCategory = 3, cex_label_category = 1.3, cex_label_gene = 1.5) + theme(text=element_text(size = 30),legend.title=element_text(size = 21), legend.text=element_text(size = 21));

png('GO-Biological_Process_netplot.png', width = 3700, height = 2000, res = 200);

print(c);

dev.off()

*# RIDGEPLOT:*

d <- ridgeplot(go_result_mf, showCategory=5) + labs(x = 'enrichment distribution') + theme(text=element_text(size = 30),legend.title=element_text(size = 30), axis.text.y=element_text(size = 30),axis.text.x=element_text(size = 30), legend.text=element_text(size = 30), axis.title.x=element_text(size = 30));

png('GO-Molecular_Function_ridgeplot.png', width = 1700, height = 2100, res = 140);

print(d);

dev.off()

d <- ridgeplot(go_result_bp, showCategory=5) + labs(x = 'enrichment distribution') + theme(text=element_text(size = 30),legend.title=element_text(size = 30), axis.text.y=element_text(size = 30),axis.text.x=element_text(size = 30), legend.text=element_text(size = 30), axis.title.x=element_text(size = 30));

png('GO-Biological_Process_ridgeplot.png', width = 1700, height = 2100, res = 140);

print(d);

dev.off()

**Figure 4.**
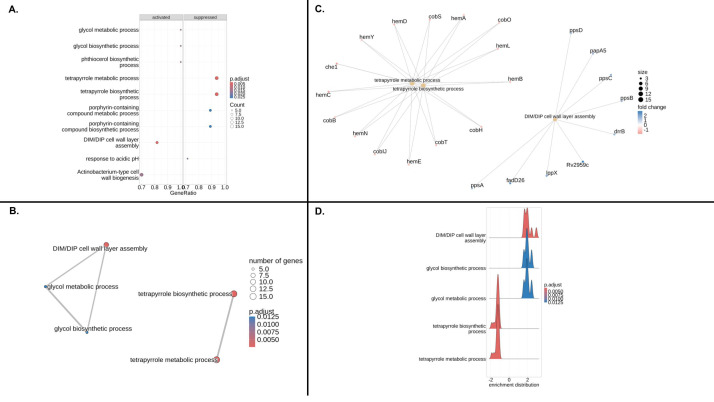
Gene ontology enrichment analysis of differentially expressed genes (*Biological Process*). (A) Dot plot showing activated and suppressed biological processes enriched among differentially expressed genes. (B) Enrichment map illustrating associations between enriched biological process terms. (C) Network plot (netplot) depicting differentially expressed genes linked to their respective enriched processes. (D) Ridge plot showing the distribution and significance of selected enriched biological processes based on adjusted p-values.

**Figure 5.**
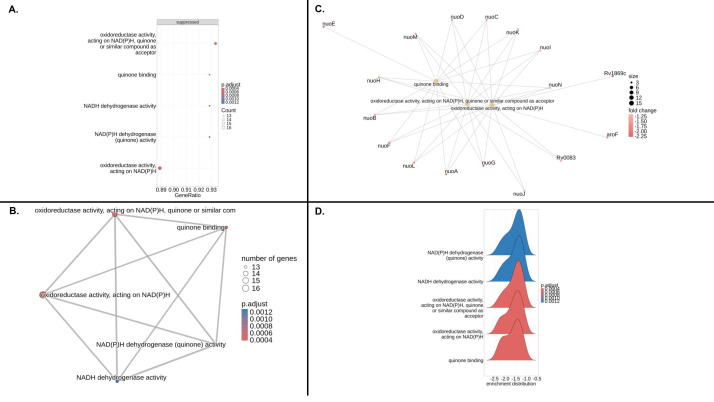
Gene ontology enrichment results (*Molecular Function*). (A) Dot plot depicting differentially expressed genes enriched and suppressed across *Molecular Function* terms. The X-axis represents the gene ratio, with dot size indicating gene count and color scale showing adjusted p-values. (B) Enrichment map illustrating functional relationships among significantly enriched *Molecular Function* terms, where edge thickness represents gene overlap. (C) Network plot showing differentially expressed genes mapped to their associated enriched *Molecular Function* terms; node size reflects gene count, and color indicates fold-change. (D) Ridge plot representing the distribution of enrichment significance across *Molecular Function* terms, colored by adjusted p-values.

Figures 4 and 5 represent the significantly enriched gene sets and enriched gene ontology components for the gene ontology terms *Biological Process* and *Molecular Function*, respectively.

To perform gene ontology and KEGG pathway enrichment analyses, use the top differentially expressed genes. Among the upregulated genes, significant over-enrichment is observed for *Biological Process* terms such as DIM/DIP cell wall layer assembly, Actinobacterium-type cell wall biogenesis, organic hydroxy compound biosynthetic process, glycol metabolic process, and glycol biosynthetic process. Conversely, the downregulated genes are strongly under-enriched for tetrapyrrole metabolic process, tetrapyrrole biosynthetic process, porphyrin-containing compound metabolic and biosynthetic processes, and response to acidic pH. *Molecular Function* analysis revealed no significant over-enrichment among the upregulated genes; however, the downregulated genes are highly under-enriched for quinone binding, oxidoreductase activity acting on NAD(P)H, oxidoreductase activity acting on NAD(P)H with quinone or similar compound as acceptor, NADH dehydrogenase activity, and NAD(P)H dehydrogenase (quinone) activity. All the enriched terms are based on significant adjusted p-values (padj < 0.05).

The results shown in Figures 4A–D and 5A–D represent only a snapshot of the overall findings. Users can generate the complete set of results by customizing the parameters in the provided scripts and visualizing them through charts.

**Figure 6.**
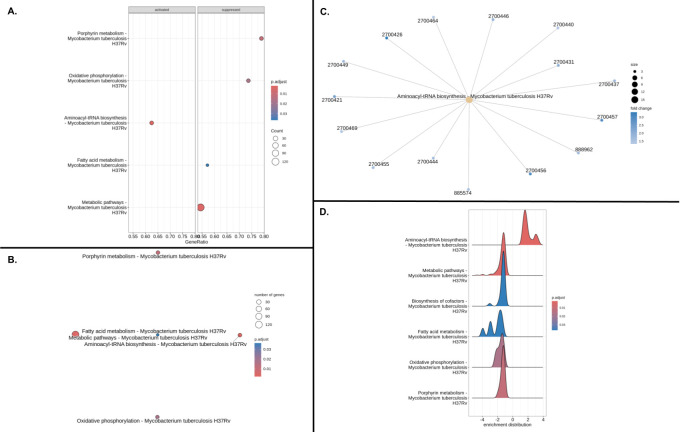
KEGG pathway enrichment analysis of differentially expressed genes in *Mycobacterium tuberculosis* H37Rv. (A) Dotplot showing significantly enriched KEGG pathways for activated and suppressed genes, with dot size representing the number of genes and color indicating adjusted p-value. (B) Enrichment map displaying the relationship among enriched KEGG pathways, highlighting clusters of functionally related pathways. (C) Network plot (netplot) illustrating differentially expressed genes associated with each enriched KEGG pathway. (D) Ridge plot showing the distribution of enrichment scores for top KEGG pathways, with color representing adjusted p-values.

After performing gene ontology analysis, pathway enrichment analysis is performed using KEGG annotations. The results from KEGG analysis are shown in Figure 6A–D, which represents the snapshot of overall findings. Users can generate the complete set of results by customizing the parameters using the provided scripts and visualizing them through charts.

*# Convert the gene type from ‘SYMBOL’ to NCBI ENTREZID ids by mapping with the org.Mtuberculosis*H37R*v.eg.db annotation package:*

ids<-bitr(names(original_gene_list), fromType = 'SYMBOL', toType = 'ENTREZID', OrgDb=org.MtuberculosisH37Rv.eg.db)

*# Remove any duplicated ids:*

dedup_ids = ids[!duplicated(ids[c('SYMBOL')]),]

*# Create a dataframe by matching the deduplicated id rows with the list of differentially expressed genes:*

df2 = input[row.names(input) %in% dedup_ids$'SYMBOL',]

*# Add another additional column of ENTREZ ids to the above filtered dataframe:*

df2$Y = dedup_ids$ENTREZID

*# Extract log2Foldchange information for the differentially expresesed genes:*

kegg_gene_list <- df2$log2FoldChange

*# Now, map the ENTREZ ids with the extracted log2FoldChanges:*

names(kegg_gene_list) <- df2$Y

*# Omit any ‘NA’ rows from the created list:*

kegg_gene_list<-na.omit(kegg_gene_list)

*# Sort the list in decreasing order:*

kegg_gene_list = sort(kegg_gene_list, decreasing = TRUE)

*# Get your organism’s three-letter code from the below website: https://www.genome.jp/kegg/tables/br08606.html*

*# For Mycobacterium tuberculosis* H37Rv, *the three-letter code is ‘mtu’*

kegg_organism <- 'mtu'

*# Now, we will perform KEGG Pathway analysis for the significantly expressed genes:*

kegg_result <- gseKEGG(geneList = kegg_gene_list, organism = kegg_organism, minGSSize = 3, maxGSSize = 800, pvalueCutoff = 0.05, pAdjustMethod = 'BH', keyType = 'ncbi-geneid')

*# Save the KEGG results in a .tsv file:*

write.table(kegg_result@result,'KEGG_results.tsv')

*# Now plot charts for visualising KEGG Pathway results:*

*# DOTPLOT:*

a <- dotplot(kegg_result, showCategory = 10, split='.sign') + facet_grid(.~.sign);

jpeg('KEGG_enrichment_dotplot.jpeg', width = 1500, height = 1000, res = 140);

print(a);

dev.off()

*# ENRICHMENT MAP:*

x2 <- pairwise_termsim(kegg_result);

b <- emapplot(x2, showCategory = 10);

jpeg('KEGG_enrichmentmap.jpeg', width = 1500, height = 1000, res = 140);

print(b);

dev.off()

*# NETPLOT:*

c <- cnetplot(kegg_result,categorySize = 'pvalue',foldChange = kegg_gene_list, showCategory = 3);

jpeg('KEGG_enrichment_netplot.jpeg', width = 3500, height = 2000, res = 200);

print(c);

dev.off()

*# RIDGEPLOT:*

d <- ridgeplot(kegg_result) + labs(x = 'enrichment distribution');

jpeg('KEGG_enrichment_ridgeplot.jpeg', width = 1500, height = 2000, res = 140);

print(d);

dev.off()

The top upregulated genes are predominantly enriched in the KEGG pathway Aminoacyl-tRNA biosynthesis. In contrast, the top downregulated genes are significantly enriched in metabolic pathways, porphyrin metabolism, oxidative phosphorylation, fatty acid metabolism, and biosynthesis of cofactors.

## Validation of protocol

The customization of annotation packages for non-model organisms using the AnnotationForge R package was adapted from the Bioconductor vignette [12]. Further refinements, including the integration of additional NCBI repositories into SQLite, were derived from best practices discussed on the Bioconductor support forum [13]. While this specific implementation has not yet been featured in a published study, the underlying methods are based on widely accepted, community-validated Bioconductor resources. These approaches have been developed and maintained by a global network of researchers and software developers, ensuring that they remain open-source, reproducible, and robust for enrichment analysis in non-model organisms. This protocol consolidates and systematizes those approaches into a single, practical workflow for broader use. The differential expression and enrichment analyses presented here are included solely to illustrate the applicability of the described workflow. The results are dependent on the dataset, chosen thresholds, statistical settings, and database versions, and should not be interpreted as definitive biological findings. Users are encouraged to adjust parameters (e.g., filtering criteria, log2FC cutoffs, statistical models, annotation sources) according to their specific experimental design, data, and research objectives.

## General notes and troubleshooting

**General notes**

1. In this protocol, the same methodology and steps described in our previous Bio-protocol [15] were followed for primary transcriptome analysis, which includes initial quality control, adapter trimming, mapping reads to the reference genome, and read counts quantification.

2. The DEG analysis section is discussed in detail, outlining essential dos and don’ts to ensure a reliable, error-free analysis. Preparing the read count matrix and metadata files is a critical step, as these must adhere to specific formatting requirements.

3. The read count file is a matrix where row names correspond to gene names and column names correspond to sample IDs or sample names. Each cell contains the number of reads mapped to a given gene in a particular sample.

4. The metadata file is a tab-separated text file containing information about each sample and its associated treatment or condition.

5. It is essential to ensure that the sample names in the metadata file match exactly, in both order and spelling, the column names in the read count matrix. Additionally, only a single tab must separate columns in the metadata file, and the file should not contain any extra white spaces or newline characters.

6. All files downloaded from NCBI and ExPASy for constructing the customized annotation package should be stored in the same folder before starting the build process.

7. In addition to the primary files used for construction of the annotation package, additional files available in the NCBI repository (https://ftp.ncbi.nlm.nih.gov/gene/DATA/) can be incorporated to enrich the customized organism annotation package.

8. Some codes given in this Bio-protocol are longer to fit into a single line. When users copy every line of these codes all at once, the lines may break and result in errors. For example, the featureCounts command is a single-line command split into multiple lines. Here, users should avoid copying every lines at once. Instead, they can copy each line of this command and paste on the console/terminal separated by spaces before running.

9. The following details on the R sessionInfo() will be useful, as they list the working environment information:

R version 4.3.3 (2024-02-29)

Platform: x86_64-conda-linux-gnu (64-bit)

Running under: Ubuntu 20.04.6 LTS

time zone: Asia/Kolkata

tzcode source: system (glibc)

attached base packages:

[1] stats4 stats graphics grDevices utils datasets methods

[8] base

other attached packages:

[1] ggplot2_4.0.0    ggridges_0.5.7

[3] enrichplot_1.22.0   clusterProfiler_4.10.0

[5] org.MtuberculosisH37Rv.eg.db_0.1  AnnotationDbi_1.64.1

[7] IRanges_2.36.0   S4Vectors_0.40.2

[9] Biobase_2.62.0   BiocGenerics_0.48.1

loaded via a namespace (and not attached):

[1] DBI_1.2.3   bitops_1.0-9  gson_0.1.0

[4] shadowtext_0.1.6  gridExtra_2.3  rlang_1.1.6

[7] magrittr_2.0.4   DOSE_3.28.1  compiler_4.3.3

[10] RSQLite_2.4.3  systemfonts_1.2.3  png_0.1-8

[13] vctrs_0.6.5   reshape2_1.4.4  stringr_1.5.2

[16] pkgconfig_2.0.3  crayon_1.5.3  fastmap_1.2.0

[19] XVector_0.42.0  labeling_0.4.3  ggraph_2.2.1

[22] HDO.db_0.99.1  purrr_1.1.0  bit_4.6.0

[25] zlibbioc_1.48.2  cachem_1.1.0  aplot_0.2.9

[28] GenomeInfoDb_1.38.8  jsonlite_2.0.0  blob_1.2.4

[31] uuid_1.2-1   BiocParallel_1.36.0  tweenr_2.0.3

[34] parallel_4.3.3   R6_2.6.1   stringi_1.8.7

[37] RColorBrewer_1.1-3  GOSemSim_2.28.0 Rcpp_1.1.0

[40] Matrix_1.6-5   splines_4.3.3  igraph_2.1.4

[43] tidyselect_1.2.1  qvalue_2.34.0  viridis_0.6.5

[46] codetools_0.2-20  lattice_0.22-7   tibble_3.3.0

[49] plyr_1.8.9   treeio_1.26.0  withr_3.0.2

[52] KEGGREST_1.42.0  S7_0.2.0   gridGraphics_0.5-1

[55] scatterpie_0.2.6  polyclip_1.10-7  Biostrings_2.70.3

[58] pillar_1.11.1   ggtree_3.10.0  ggfun_0.2.0

[61] generics_0.1.4  RCurl_1.98-1.17  scales_1.4.0

[64] tidytree_0.4.6   glue_1.8.0  lazyeval_0.2.2

[67] tools_4.3.3   ggnewscale_0.5.2  data.table_1.17.8

[70] fgsea_1.28.0   ggiraph_0.8.12  fs_1.6.6

[73] graphlayouts_1.2.2  fastmatch_1.1-6  tidygraph_1.3.0

[76] cowplot_1.2.0  grid_4.3.3  tidyr_1.3.1

[79] ape_5.8-1    colorspace_2.1-2  nlme_3.1-168

[82] GenomeInfoDbData_1.2.11 patchwork_1.3.2  ggforce_0.5.0

[85] cli_3.6.5   rappdirs_0.3.3  viridisLite_0.4.2

[88] dplyr_1.1.4   gtable_0.3.6  yulab.utils_0.2.1

[91] digest_0.6.37   ggrepel_0.9.6  ggplotify_0.1.3

[94] htmlwidgets_1.6.4  farver_2.1.2  htmltools_0.5.8.1

[97] memoise_2.0.1  lifecycle_1.0.4  httr_1.4.7

[100] GO.db_3.18.0  bit64_4.6.0-1  MASS_7.3-60.0.1

10. The Bio-protocol mainly focuses on performing differential expression analysis and gene enrichment analysis using RNA-Seq datasets, particularly highlighting how gene enrichment can be performed for any other non-model organism or under-annotated species using customized annotation R package. Users can follow all analysis methods described in the Bio-protocol, from performing initial QC to differential expression analysis. While using the command for constructing the customized annotation package, users have to replace the genus name, species name, and taxonomic identifier that matches their species of interest. Every time the user creates a customized annotation package for an organism, the package for that organism is created inside a folder that ends with the name .eg.db. Users can install the package into the R environment using the following command:

install.packages("./org.example.*eg.db*", repos=NULL)

The annotation package folder has to be found in the current working directory before installing the package. This is a one-time installation. Later, installed packages can be simply loaded as R libraries and can be used with clusterProfiler to perform gene enrichment analysis.

11. The dataset used in this manuscript focuses on a particular strain, *Mycobacterium tuberculosis* H37Rv, since this protocol is published from a research institute that mainly focuses on tuberculosis research. Besides that, the protocol aims to demonstrate the analysis methods using the publicly available datasets on this strain, for which we have selected the RNA-Seq dataset “GSE292409.” We could observe higher variability in the sequencing depth of this dataset within replicates. Though the percentage of duplicate reads is expected to be high in RNA-Seq datasets, we might observe high variability when we sequence smaller transcriptomes and when subjected to certain treatments or conditions. In order to mitigate this limiting factor, we have used quality control methods like adapter trimming, mapping reads to reference genome, clustering samples by PCA, and normalization methods by DESeq2. These methods ensure that only high-quality reads pass through downstream analysis. Apart from the dataset used above, the methods used in the protocol can be applied to any of the RNA-Seq datasets.

**Troubleshooting**

**Problem 1:** Killing of the process during the generation of the customized annotation package.

Possible cause: Database connectivity issues, as some steps require direct retrieval of data from FTP sites.

Solution: To prevent this, ensure a stable and uninterrupted internet connection and verify that FTP access is not blocked by your firewall.

**Problem 2:** Software installation and usage errors.

Possible causes: Version incompatibility, dependency issues, outdated parameters in older versions of software.

Solution: Before starting the analysis, confirm that all required R packages are installed via Bioconductor or Conda. Some enrichment plots may also require additional tools for customization. If any parameters in an R package become outdated, update the necessary dependencies to resolve errors.

**Problem 3:** Empty results during enrichment analysis.

Possible cause: No enrichment would be performed in some cases for certain sets of genes.

Solution: When performing gene ontology or KEGG pathway analysis, verify whether any significantly enriched terms are detected by running the following command:

*go_result@result$ID*

*# If the result is NULL, then no enrichment terms are found.*

*# Similarly, for KEGG, use*

*kegg_result@result$ID*

No enrichment will be performed in these cases, and hence, results plots will also not be generated.

To obtain meaningful results, it is important to generate sequencing data with sufficient depth and, where possible, increase the number of biological replicates for each condition. In some cases, enrichment analysis may fail to yield significant results due to the fold-change cutoff applied. Adjusting this threshold can help identify a greater number of enriched terms.
